# The Life of the Trichina

**Published:** 1869-05

**Authors:** 


*The Life of the Trichina.*—By Rudolph Virchow, M.D.,Ph.D. Professor University of Berlin. Translated by Prof. Rufus King Browne, M.D. A pamphlet of forty-eight pages, containing a full description of this microscopical parasite. Commencing with the history of the trichina this work investigates the following queries: How do we recognize trichinæ in meat? What dangers to the human body do the trichinæ cause? What preventive measures against its spread are advisable? The three cardinal points elucidated are: 1. The eaten Trichinæ remain in the intestine, and never enter the muscles. 2. They produce living young, which enter the muscles. 3. The young which have entered the muscles grow there but do not multiply. The main danger, therefore, is in the production of young by the intestinal Trichina.

